# Current News

**Published:** 1890-04

**Authors:** 


### Current News.

### CHICAGO AND VICINITY.

Dr. E. S. Talbot, whose labors in the field of Dental Irregularities are well known, has made a thorough and scientific examination of the mouths of the "cave and cliff dwellers," who have been on exhibition under the charge of Lieutenant Schwatka, and states that the results were interesting and instructive. They will be given to the profession later on.

Mr. E. E. Clark, of Newark, N. J., has been in the city, arranging matters with a view of establishing a plant for the manufacture of his deposited plates in this city. A number of prominent dentists appear to have taken an interest in the matter.

The Chicago Dental Club appears to be in a flourishing and growing condition, and arrangements have been made for publishing its proceedings, in future, in the International Dental Journal.

Dr. Custer, Dayton Ohio, says too much value cannot be placed upon the power of personal magnetism in the dental operator. The exhibition of tender sympathy in a painful operation does much to mitigate its severity. Everything lies in obtaining the absolute confidence of the patient. If the operator shows that he is perfectly familiar with the operation, that he knows exactly what to do, giving no evidence of bungling or embarrassment, no hesitation in the choice of instrument or remedy, there will be less dread and apprehension, the imagination will not be roused, and, the subjective image not being formed, the actual will not be realized.

Dr. Green, New Albany, suggests that, in setting a porcelain inlay in cement, the piece of tooth be made as hot as can be held in the fingers before pressing home in the cement. The cement will set much harder.

Chloride of zinc stands at the head of the list as an obtundant of sensitive dentine. It both coagulates and dehydrates, but its action is painful, and, as it is not self-limiting, it must be used with caution.

---

Dr. A. E. Baldwin, Chicago, overcomes sensitive dentine by the use of sure, sharp instruments, with rapid motion and light touch.

---

### SOCIETY NOTICES.

The Iowa State Dental Society will hold its Twenty-eighth Annual Meeting at Dubuque, Iowa, May 6 to 9, 1890. All are invited to attend.

---

### PROGRAMME OF DENTAL SECTION OF THE AMERICAN MEDICAL ASSOCIATION.

Address. By *J. L. Williams.*

Relation of Tropho-Neuroses to Diseases of the Mouth and Jaws, with Special Reference to Syphilitic Necrosis. By *G. Frank Lydston.*

How the Vascular Supply is connected with the Teeth. By *A. O. Hunt.*

Vascular Tumors of the Mouth, and Treatment by Injection. By *John Marshall.*

Electro-Therapeutics. By *John L. Gish.*

The Value of Illustration in the Lecture-Room. By *L. D. McIntosh.*

Adenoid Growths and their Effect on the Mouth. By *E. E. Briggs.*

Cure for Cleft Palate by a Double-Flap Operation and Closure with the buried Tendon Suture. By *H. O. Marcy.*

Diseases of the Gums and their Treatment. By *J. Taft.*

Irregularities of the Teeth connected with Neurotic Conditions. By *E. S. Talbot.*

Hereditary Dental Anomalies. By *William S. Sherman.*

